# Developing the Oxalate, Fumarate and Succinate Salts of Tetrabenazine: Solid-State Characterization and Solubility

**DOI:** 10.3390/pharmaceutics17050670

**Published:** 2025-05-20

**Authors:** Marieta Muresan-Pop, Viorica Simon, Gheorghe Borodi, Alexandru Turza

**Affiliations:** 1 Interdisciplinary Research Institute in Bio-Nano-Sciences, Contrast Agents and Specific Therapeutics Center—INSPIRE Platform, Babes-Bolyai University, 42 Treboniu Laurian, 400271 Cluj-Napoca, Romania; 2 Faculty of Physics, Babes-Bolyai University, 1, Kogalniceanu, 400084 Cluj-Napoca, Romania; viorica.simon@ubbcluj.ro; 3 National Institute for R&D of Isotopic and Molecular Technologies, 67-103 Donat, 400293 Cluj-Napoca, Romania; borodi@itim-cj.ro

**Keywords:** tetrabenazine, solid forms, ball milling, crystal structure, supramolecular interactions, solubility enhancement, salt

## Abstract

**Background**: Tetrabenazine (brand name Nitoman and Xenazine) is a compound used to treat neurological and psychiatric disorders. Due to its low solubility, this drug is administered to patients in high doses, which produces side effects. **Methods**: To overcome these deficiencies, we prepared, using the mechanochemical method, three salts of tetrabenazine with three coformers: oxalic, fumaric, and succinic acid. The new solid forms were identified by X-ray powder diffraction (XRPD). **Results**: Full structural characterization was performed by single-crystal X-ray diffraction (SC-XRD), which revealed that the supramolecular interactions in the new solid forms were achieved by proton transfer between the coformer and the nitrogen of the tetrabenazine molecule. The salts formation was also evidenced by thermal analyses (DSC) and infrared spectroscopy (FTIR). Furthermore, the physical stability of the salts was evaluated under extreme temperature and humidity conditions. **Conclusions**: From a pharmaceutical perspective, UV-VIS tests of the new salts dissolved in water revealed a significant improvement in their solubility, which could improve their bioavailability in therapeutic applications.

## 1. Introduction

Tetrabenazine (C_19_H_27_NO_3_), also known by its IUPAC chemical name 3-isobutyl-9,10-dimethoxy-1,3,4,6,7,11b-hexahydro-2H-pyrido[2,1-a]isoquinolin-2-one, is a synthetic compound classified as a dopamine depleter [1]. It is primarily used to treat neurological disorders and psychiatric disturbances [2]. Notably, tetrabenazine is the only FDA-approved drug for the management of chorea associated with Huntington’s disease [3,4], a condition that helps manage the depletion of dopamine through reversible inhibition of the human vesicular monoamine transporter 2 (VMAT2) [5].

The dosage of tetrabenazine varies based on patient tolerance and therapeutic needs, typically ranging from 12.5 mg to 100 mg per day. However, it can cause several adverse effects, including akathisia, restlessness, parkinsonism, depression, insomnia, anxiety, and intolerable sedation [6]. These side effects underscore the need for the development of new tetrabenazine formulations with enhanced solubility and potentially fewer adverse reactions. Various methods are used to improve the physicochemical properties and bioavailability of active pharmaceutical ingredients, as complexation with beta-cyclodextrin [7,8,9], and the development of new solid forms (ex. polymorphs, cocrystals, salts, and solvates) [10,11]. The obtainment of new crystalline forms of a pharmaceutical involves hydrogen bonds, π···π interactions, and van der Waals contacts [12,13] to enable different packing within the crystal [14]. For example, cocrystals and solvates involve the formation of a new multi-component compound that includes at least one guest molecule in addition to a host molecule, and, usually in salt form, proton transfer occurs from the guest molecule to the host molecule.

In the literature, there are studies on various forms of tetrabenazine, including anhydrous tetrabenazine [15], dihydrotetrabenazine [16], (+)-tetrabenazine (1S)-(+)-10-camphorsulfonate monohydrate [17], and various tetrabenazine derivatives [18]. Despite these efforts, there remains a need for new solid forms of tetrabenazine that offer improved pharmacological properties.

The objective of this study is to prepare novel tetrabenazine-based materials in the form of salts via mechanochemistry, which present enhanced solubility. Mechanochemistry is known to be a suitable method for the preparation of such materials [19]. Various pharmaceutical agents were previously prepared as salts and cocrystals using the mechanochemistry method, such as vinpocetine and vincamine [20,21], praziquantel [22], and etravirine [23]. In this paper, we successfully report the synthesis of three new tetrabenazine salts: oxalate, fumarate, and succinate. These salts were characterized using a variety of techniques, including single-crystal X-ray diffraction, powder X-ray diffraction (PXRD), Fourier-transform infrared (FTIR) analysis, differential scanning calorimetry (DSC), and UV-VIS spectroscopy to explore their enhancement in solubility.

## 2. Materials and Methods

Tetrabenazine (317.42 g/mol, purity 98%) was provided by Hangzhou DayangChem Co., Ltd. (Hangzhou, China). Carboxylic acids: oxalic acid (126.07 g/mol), fumaric acid (116.072 g/mol), and succinic acid (118.08 g/mol) were supplied by AlfaAesar. The analytical grade of the solvents used were obtained from Sigma-Aldrich (St. Louis, MO, USA). All reagents and solvents were of analytical grade.

### 2.1. Tetrabenazine Salts Preparation and Single Crystal Growth

Co-crystallization experiments were conducted by mechanically grinding mixtures of tetrabenazine (0.315 mmol) and the selected carboxylic acid in a ball mill. Equal amounts of tetrabenazine and acid were mixed with a small quantity of solvent, and grinding was performed for 60 min at a frequency of 30 Hz. The solvent was added dropwise, with a 10 min break between two grinding cycles. The solvents used were as follows: 500 µL methanol/water (8:2 *v*/*v*) mixture for the fumaric acid (TBZ&FUM) and oxalic acid (TBZ&OXa) combinations and 400 µL tetrahydrofuran/2-propanol (2:1 *v*/*v*) mixture for the succinic acid (TBZ&SUC) combination. In the case of using oxalic acid as a coformer, a second TBZ&OXb sample was prepared, using acetonitrile as the wetting solvent, and a milling time of 150 min. After grinding, single crystals suitable for X-ray analysis were grown from saturated solutions of the same solvent, which were kept in the refrigerator for up to three weeks to allow for slow evaporation. In the case of recrystallization of the two samples prepared with oxalic acid, single crystals of acceptable sizes for single-crystal X-ray diffraction were obtained only in the case of recrystallization of the TBZ&OXb sample in acetonitrile. As a result, the crystal and molecular structure were reported for this form, with the notation TBZ&OX.

**Figure 1:**
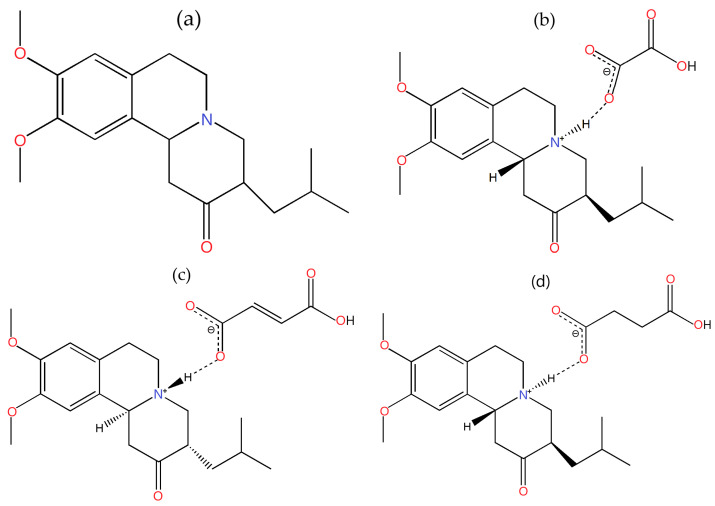
Detailed structures of tetrabenazine and the investigated salts: tetrabenazine (**a**), tetrabenazine oxalate (**b**), tetrabenazine fumarate (**c**), and tetrabenazine succinate (**d**).

The molecular scheme of tetrabenazine and the salts prepared are listed below:

(i) Tetrabenazine (denoted TBZ, Figure 1a);
(ii) The oxalate salt of tetrabenazine (denoted TBZ&OX, Figure 1b);
(iii) The fumarate salt of tetrabenazine (denoted TBZ&FUM, Figure 1c);
(iv) The succinate salt of tetrabenazine (denoted TBZ&SUC, Figure 1d).

### 2.2. Single-Crystal X-Ray Diffraction

Single crystals of the obtained salts through recrystallization were mounted on a dual microsource, SuperNova Cu at home/near, Eos diffractometer (Rigaku, Tokyo, Japan). Data were collected at 293(2) K using ω scans with Cu Kα radiation. The structures were solved with ShelXT [24] by Intrinsic Phasing and refined using ShelXL [25], which was implemented in Olex2 (version 1.2.10, Durham, UK) [26] as the graphical interface. The diffraction pattern was indexed, and the unit cell was refined using CrysAlisPro (version 40_64.84a, Yarnton, Oxfordshire, UK) [27], with data reduction, scaling, and absorption corrections being applied. Absorption corrections were performed using spherical harmonics and the SCALE3 ABSPACK scaling algorithm.

H atoms attached to carbon atoms were located, treated, and refined as riding atoms, with the isotropic displacement parameter U_iso_(H) set to 1.2 Ueq(C) for ternary CH groups [C-H = 0.93 Å], secondary CH_2_ groups [C-H = 0.97 Å], and 1.5 Ueq(C) for all methyl (CH_3_) groups [C-H = 0.96 Å]. The H atoms bonded to protonated N atoms were located through Fourier maps and refined with the bond lengths of N-H = 0.96 Å.

### 2.3. X-Ray Powder Diffraction

X-ray powder patterns were recorded with a Shimadzu 6000 Diffractometer (Shimadzu Corporation, Kyoto, Japan), equipped with a Ni (1,1,1) monochromator placed in front of the detector. Diffractograms were collected in Bragg–Brentano geometry, the reflection variant, in the angular range 2Ɵ: 4–40°, with a step of 0.01°.

### 2.4. Differential Thermal Analysis

Differential scanning calorimetry (DSC) was conducted using a DSC-60 thermal analyzer (Shimadzu Corporation, Kyoto, Japan). The samples were placed in closed aluminum crucibles with perforated lids. The measurements were carried out in a nitrogen atmosphere with a flow rate of 70 mL/min. A heating rate of 10 °C/min was maintained throughout the experiments, and Al_2_O_3_ was used as the reference sample.

### 2.5. Fourier-Transform Infrared Spectroscopy

FTIR measurements were performed with a Jasco 6200 FTIR spectrometer (JASCO Corporation 2967-5, Ishikawa-machi, Hachioji-shi, Tokyo, 192-8537, Japan). The investigated samples were prepared in the form of KBr pellets and measured in the spectral range 4000–400 cm^−1^, with a resolution of 4 cm^−1^ and 256 scans.

### 2.6. Stability

The stability assessment was performed using the Memmert Humidity Chamber HCP105 (Memmert Central Europe s.r.o. Bratislava, Slovakia), which offers precise control over both relative humidity (with an accuracy of ±1% RH) and temperature (with an accuracy of ±0.1 °C). Samples were periodically collected from the chamber and analyzed through X-ray powder diffraction to detect any potential structural changes.

### 2.7. UV Measurements

In vitro, solubility tests were conducted using the Jasco V-650 Spectrometer (Jasco Corporation, Ishikawa-machi, Hachioji-shi, Tokyo, Japan) with the LV-724 integrating sphere for UV tests on liquids. UV/VIS spectra analysis was performed using JASCO V-650 Spectra Analysis version 1.16.00.

Four stock solutions were prepared by gradually adding deionized water (pH = 6) to approximately 20 mg of each compound until the substance was completely dissolved. After stirring for 24 h at 37 °C ± 1 °C on a magnetic plate (100 rpm), the solutions were filtered through a 0.45 μm syringe filter to remove any undissolved matter. The concentrations of the stock solutions, calculated from the ratio between the mass of the substance and the total volume of water used to completely dissolve it, were as follows: C_TBZ_ = 0.0888 mg/mL, pH = 6.2; C_TBZ&OX_ = 4.3478 mg/mL, pH = 5.4; C_TBZ&FUM_ = 4.2853 mg/mL, pH = 4.8; and C_TBZ&SUC_ = 1.000 mg/mL, pH = 4.7. UV analysis of the solutions was conducted over the wavelength range of 200–500 nm. A quartz cuvette with a path length of 1 cm and a volume of 500 μL was used as the support for UV analyses.

## 3. Results

### 3.1. Analysis of Crystal Structures

The single crystal diffraction data and refinement details of the three analyzed samples are displayed in Table 1. Molecular diagrams were generated using Mercury software [28], and the representative intermolecular interactions are listed in Table S1 (Supporting Information).

#### 3.1.1. TBZ&OX

**Figure 2:**
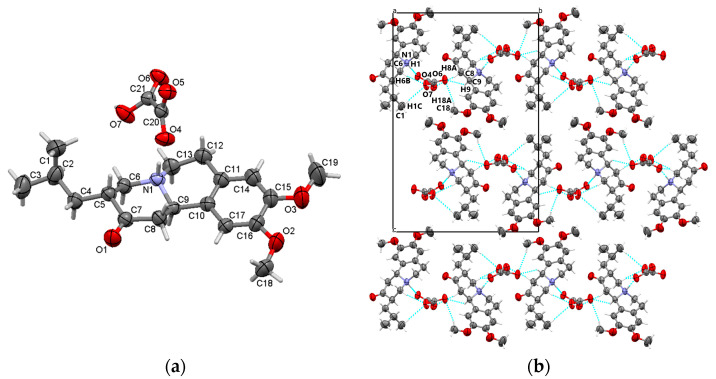
Asymmetric unit of TBZ&OX displaying the atoms as thermal ellipsoids at 50% probability (**a**); crystal packing view in the direction of the a-axis (**b**).

The analysis of the TBZ&OX by X-ray diffraction and the electron densities Fourier revealed an interesting observation: the preparation of this compound led to the formation of an oxalate salt of tetrabenazine. This is evidenced by the migration of a hydrogen atom from oxalic acid, resulting in the protonation of the nitrogen atom of tetrabenazine. The asymmetric unit of the newly formed assembly, shown in Figure 2a, consists of an oxalate anion and a tetrabenazinium cation.

In terms of solid-state stability and cohesion, the N1-H1···O4 hydrogen bonds formed between the oxalate ion and the protonated nitrogen atom (N1) of the tetrabenazine molecule play a crucial role. These hydrogen bonds are complemented by C-H···O interactions, extending in the ob direction, where the oxalate ion serves as a bridge (Figure 2b).

#### 3.1.2. TBZ&FUM

**Figure 3:**
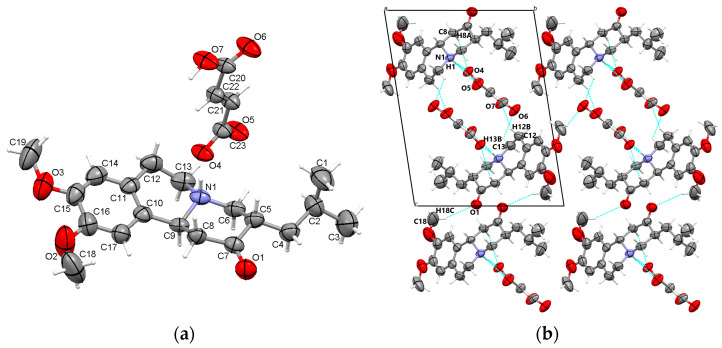
Asymmetric unit of TBZ&FUM displaying the atoms as thermal ellipsoids at 50% probability (**a**); crystal packing view in the direction of a-axis (**b**).

Similarly to the TBZ&OX crystal, X-ray analysis and Fourier map analysis of the TBZ&FUM crystal confirmed that the structure is characterized by the protonation of the nitrogen atom (N1) of the tetrabenazine molecule, resulting in the formation of a tetrabenazinium cation, while the fumarate anion undergoes deprotonation (Figure 3a). The stability of the crystal is primarily governed by N1-H1···O4 interactions between the tetrabenazinium cation and the fumarate anion. Additionally, C-H···O interactions, both between tetrabenazinium cations and between fumarate ions, further stabilize the structure, forming chains along the **a** axis (Figure 3b).

#### 3.1.3. TBZ&SUC

**Figure 4:**
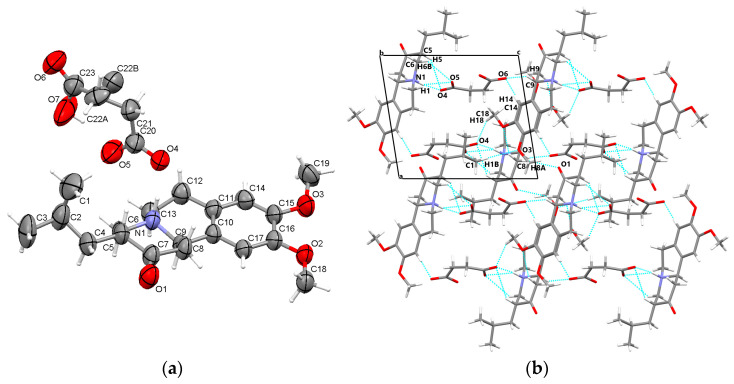
Asymmetric unit of TBZ&SUC displaying the atoms as thermal ellipsoids at 50% probability (**a**); crystal packing view in the direction of b-axis (**b**).

Similarly to the other two crystals, the TBZ&SUC crystal is formed as a salt and is characterized by the deprotonation of succinic acid, with the proton migrating to the nitrogen atom (N1) of tetrabenazine (Figure 4a). The succinate anion adopts a bent and twisted configuration, which, in contrast to the other two crystals, leads to the formation of the intramolecular hydrogen bond O7-H7···O5. Additionally, positional disorder is observed at the carbon atom C22. The protonated nitrogen atom (N1) participates in hydrogen bonding (N1-H1···O4) with the succinate ion, while other intermolecular interactions, such as C-H···O contacts, contribute to the overall crystal cohesion. These interactions occur between the carboxylate group and neighboring tetrabenazinium ions, as well as between tetrabenazinium ions themselves. The crystal packing along the b axis is presented in Figure 4b.

Single-crystal X-ray diffraction analysis concludes that the protonation of nitrogen atom of tetrabenazine molecules from the guest carboxylic acids occurs in all three crystals, providing the essential info that the newly obtained adducts are found in the form of salts instead of cocrystals and the supramolecular architectures are governed by N-H···O hydrogen bonds between the protonated nitrogen of tetrabenazinium cations with the deprotonated carboxylated anions and completed by various C-H···O and O-H···O interactions.

### 3.2. Powder X-Ray Diffraction Analysis

**Figure 5:**
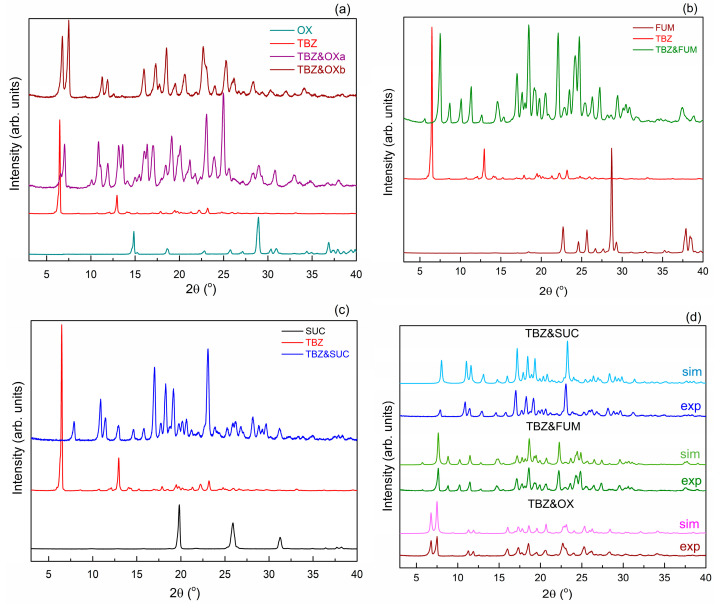
X-ray powder diffraction patterns of the new salts: TBZ&OX (**a**), TBZ&FUM (**b**), and TBZ&SUC (**c**), compared with the starting materials. The diffractograms of the new salts obtained experimentally, compared with the simulated diffractograms from the CIFs of their structures (**d**).

The diffractograms of the four samples prepared by mechanical grinding are presented in Figure 5. and compared with the initial ingredients.

The comparison of the samples obtained from tetrabenazines with oxalic acid (Figure 5a) revealed that in the case of the TBZ&OXa sample ground with a methanol/water mixture, the unreacted substance remains, only the TBZ&OXb form, ground with acetonitrile and for a longer time, is a new pure form. The difference between the two solid forms of tetrabenazines with oxalic acid indicates that both the solvent used to wet the ingredients and the time of the mortar process play an important role in the process of obtaining a new solid form. The pure form was obtained when acetonitrile was used, and a long mortar time of 150 min was used [22]. In the following, for simplicity, this form will be denoted as TBZ&OX.

Analysis of the diffractograms of the milled samples TBZ&OX, TBZ&SUC (Figure 5b), and TBZ&FUM (Figure 5c) highlights the presence of well-defined diffraction peaks, which do not correspond to the raw materials, confirming the absence of unreacted compounds. The new diffraction peaks identified in the diffraction patterns of the milled samples further validate the formation of new solid forms of tetrabenazine. Comparisons between the X-ray diffractograms of the materials obtained by the mechanochemistry method and those simulated from CIF files on single crystals confirm that the samples obtained by the mechanochemistry method and those obtained by mechanochemistry followed by recrystallization are the same (Figure 5d).

### 3.3. DSC Thermal Analysis

Differential scanning calorimetry (DSC) can be used to detect and investigate the successful preparation of new crystals, including co-crystals, salts, and solvates. This is often indicated by a melting point that differs from that of the starting material. Such changes are attributed to alterations in the intermolecular interactions and the arrangement of molecules within the crystal lattice [29,30,31,32].

#### 3.3.1. TBZ&OX

**Figure 6:**
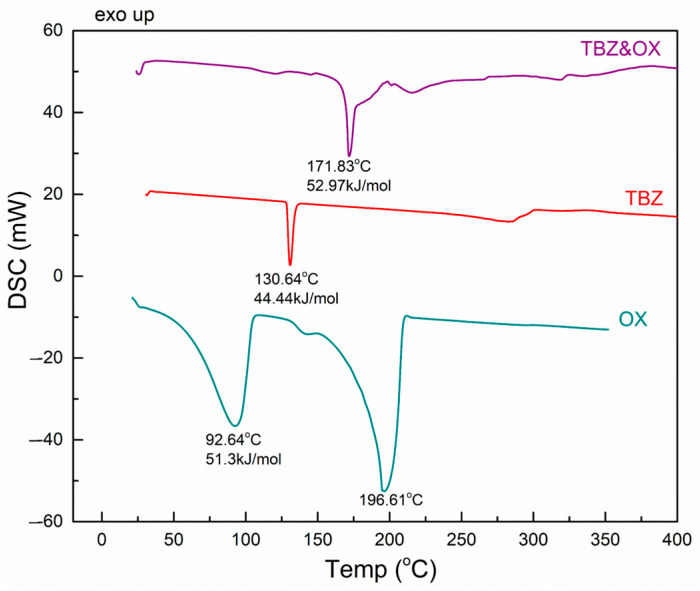
DSC thermogram of oxalic acid, tetrabenazine, and the oxalate salt of tetrabenazine.

The DSC curve of tetrabenazine (Figure 6) shows a sharp endothermic peak at approximately 130.6 °C, which corresponds to the melting point of the compound [33]. Additionally, an endothermic signal with a maximum of 282.5 °C is observed, indicating the decomposition of tetrabenazine.

The DSC diagram of oxalic acid shows a broad endothermic peak with a maximum at 92.6 °C, which is associated with the release of water molecules from the crystal lattice [34]. The subsequent endothermic peak observed at 196.6 °C corresponds to its melting point. In contrast, the thermogram of the newly prepared TBZ&OX salt exhibits an endothermic peak at 171.8 °C, indicating its melting point (Figure 6).

#### 3.3.2. TBZ&FUM

**Figure 7:**
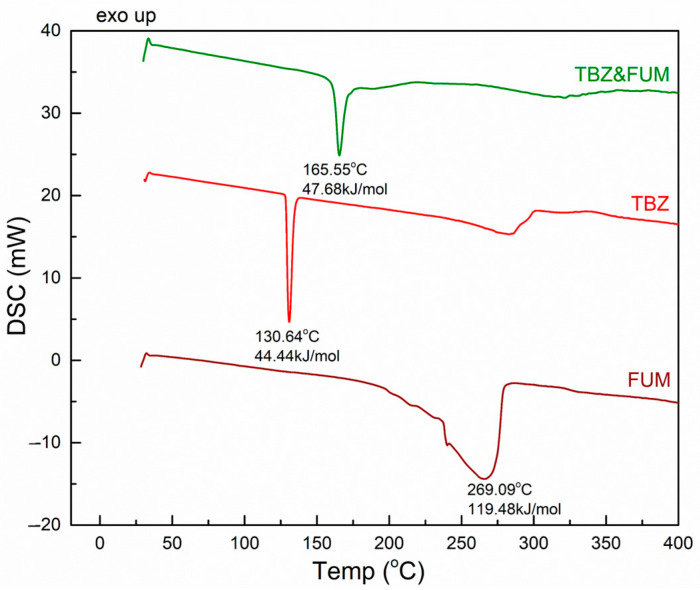
DSC thermogram of fumaric acid, tetrabenazine, and the fumarate salt of tetrabenazine.

The DSC curve of fumaric acid (Figure 7) exhibits a broad endothermic peak, starting around 200 °C, with a maximum at 269 °C, which is attributed to its melting point.

In contrast, the DSC curve of the newly prepared TBZ&FUM salt (Figure 7) displays thermal behavior that reflects its melting point, which is recorded at 165.5 °C. This value lies between the melting points of the starting materials, demonstrating the formation of a new solid form.

#### 3.3.3. TBZ&SUC

**Figure 8:**
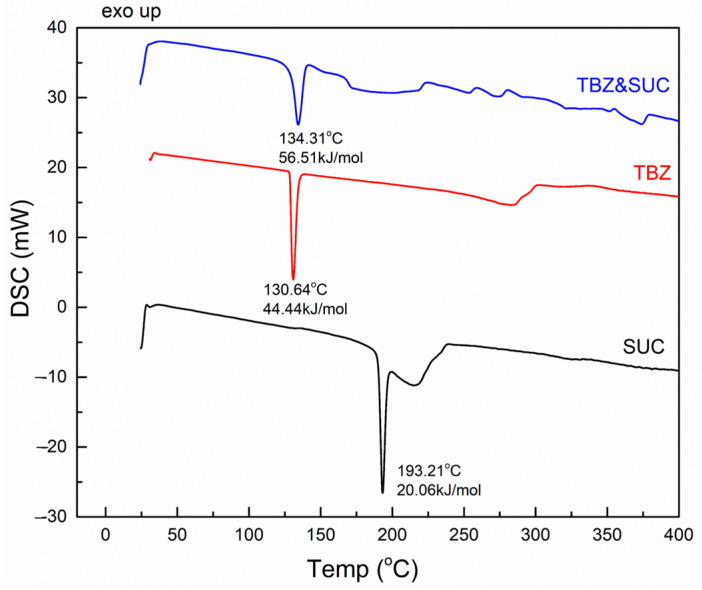
DSC thermogram of succinic acid, tetrabenazine, and the succinate salt of tetrabenazine.

The DSC thermogram of succinic acid (Figure 8) shows a sharp endothermic peak at 193.2 °C, corresponding to its melting point. Additionally, a broad endothermic peak appears at 215 °C, which is associated with decomposition.

The DSC curve of the newly obtained TBZ&SUC salt exhibits an endothermic peak at 134.3 °C, which differs from the melting points of the individual starting materials: 130.6 °C for pure tetrabenazine and 193 °C for succinic acid (Figure 8). This indicates the formation of a new solid form with a distinct melting point.

In conclusion, the DSC thermal analysis demonstrates that the newly prepared salts have distinct melting points compared to the starting materials (both tetrabenazine and the carboxylic acids used). Notably, for all three new salts, the melting point lies between those of tetrabenazine and the coformer.

### 3.4. Stability of the New Tetrabenazine-Based Salts

The instability of a particular new solid form, such as polymorphs, cocrystals, and salts, can be seen as a drawback, as phase transitions between these solid forms during prolonged storage may alter the drug’s physical state, potentially impacting its shelf life and effectiveness. Therefore, it is crucial to store the crystalline forms in a controlled environment with specific humidity and temperature conditions (75% relative humidity and 40 °C) to evaluate their stability. The new salt samples were stored for up to 20 weeks in a climatic chamber, with powder X-ray diffraction patterns recorded at four-week and twenty-week intervals.

**Figure 9:**
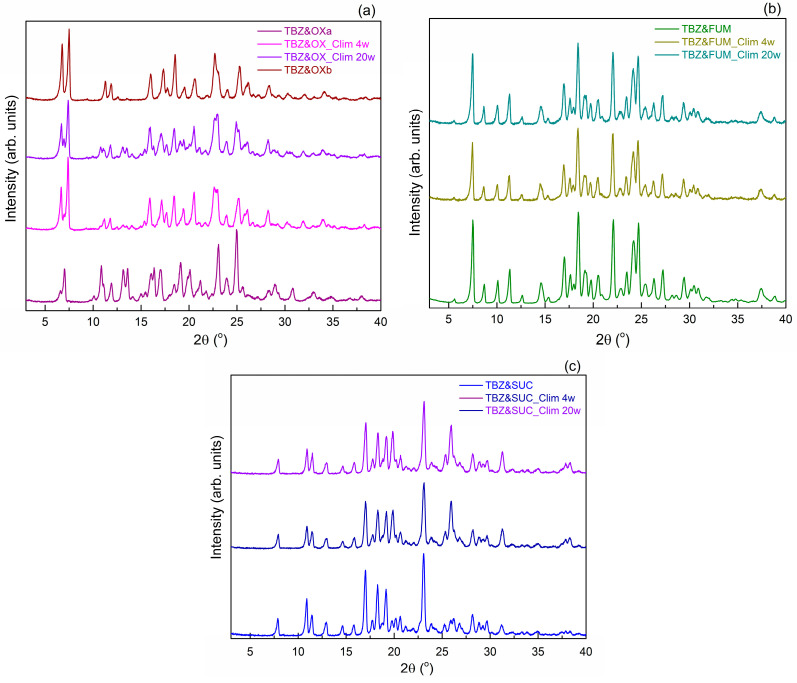
X-ray diffraction patterns comparison for the samples kept in the climatic chamber up to 20 weeks: TBZ&OX (**a**); TBZ&FUM (**b**); and TBZ&SUC (**c**).

Since the XRD analysis showed that traces of unreacted raw materials remained in the TBZ&OXa sample, this sample was kept in the climatic chamber to test whether, by storing it in extreme temperature and humidity conditions, the interaction between the ingredients continues. From the comparison of the diffractograms obtained on the TBZ&OXa sample before and after the climatic chamber, with the diffractogram of the TBZ&OXb sample (obtained with acetonitrile) (Figure 9a), it is found that the sample TBZ&OXa (obtained whit methanol/water) tends to transform into the stable form TBZ&OXb-identical to the single crystal form.

From the stability tests on the other two new solid forms TBZ&FUM and TBZ&SUC, by comparing the diffractograms of the samples before and after keeping in the climate, it resulted that they do not suffer significant structural changes during this period, which suggests that the samples remained stable and did not undergo phase transitions (Figure 9b,c).

### 3.5. FTIR Spectroscopy

Fourier-transform infrared (FTIR) spectroscopy is a valuable technique for studying new solid forms, including co-crystals, salts, and solvates. FTIR provides important insights into the molecular vibrations and functional groups within a compound, making it particularly useful for distinguishing between different solid forms.

**Figure 10:**
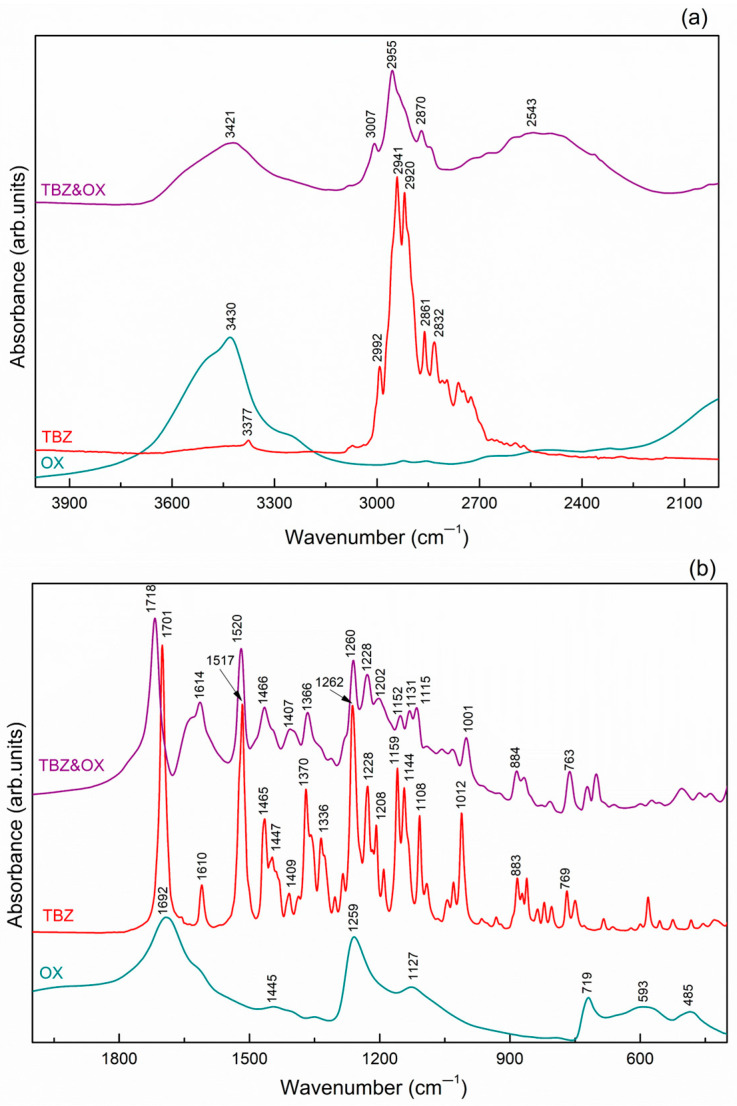
FTIR spectra comparison of oxalic acid, tetrabenazine, and the new oxalate salt of tetrabenazine in the higher frequency region (**a**) and the fingerprint region (**b**), respectively.

In the FTIR spectrum of pure tetrabenazine (Figure 10), several characteristic vibrations are observed, corresponding to the functional groups present in the molecule. At 3377 cm^−1^, a low-intensity band is detected, which is associated with the O-H stretching vibration, likely due to the presence of surface water. In the frequency range of 2992 to 2725 cm^−1^, a series of bands are observed, mainly attributed to the C-H stretching vibrations of the methyl and methylene groups in the tetrabenazine molecule [35].

The sharp absorption band located at 1701 cm^−1^ in the TBZ spectrum can be assigned to the C=O carbonyl group stretching vibration [14]. Additionally, the C=C stretching vibration from the benzene ring is observed between 1610, 1517, and 1465 cm^−1^ [14]. The stretching vibration of the amine group C-N is observed at 1228 cm^−1^, and the vibration at 1262 and 1108 cm^−1^ is associated with the stretching vibration of the aromatic ether C-O-C [4,35].

#### 3.5.1. TBZ&OX

The FTIR spectrum of oxalic acid (Figure 10) displays a broad band at 3430 cm^−1^, which can be attributed to the O-H vibrations of both water molecules incorporated in the network and the carboxyl groups. The wavenumber at 1692 cm^−1^ corresponds to the C=O stretching vibration of the carboxyl group.

In the spectrum of the TBZ&OX salt (Figure 10), the O-H vibrations characteristic of the oxalate anion is observed at 3421 cm^−1^. The stretching bands of the C-H groups are identified between 3007 and 2870 cm^−1^. The protonation of the nitrogen atom in the tetrabenazine molecule is highlighted by a broad band at 2543 cm^−1^, which corresponds to the N-H group. The C=O stretching vibration of the newly formed complex is observed at 1718 cm^−1^, while the C=C stretching vibrations in the aromatic rings appear at 1614 and 1520 cm^−1^.

#### 3.5.2. TBZ&FUM

**Figure 11:**
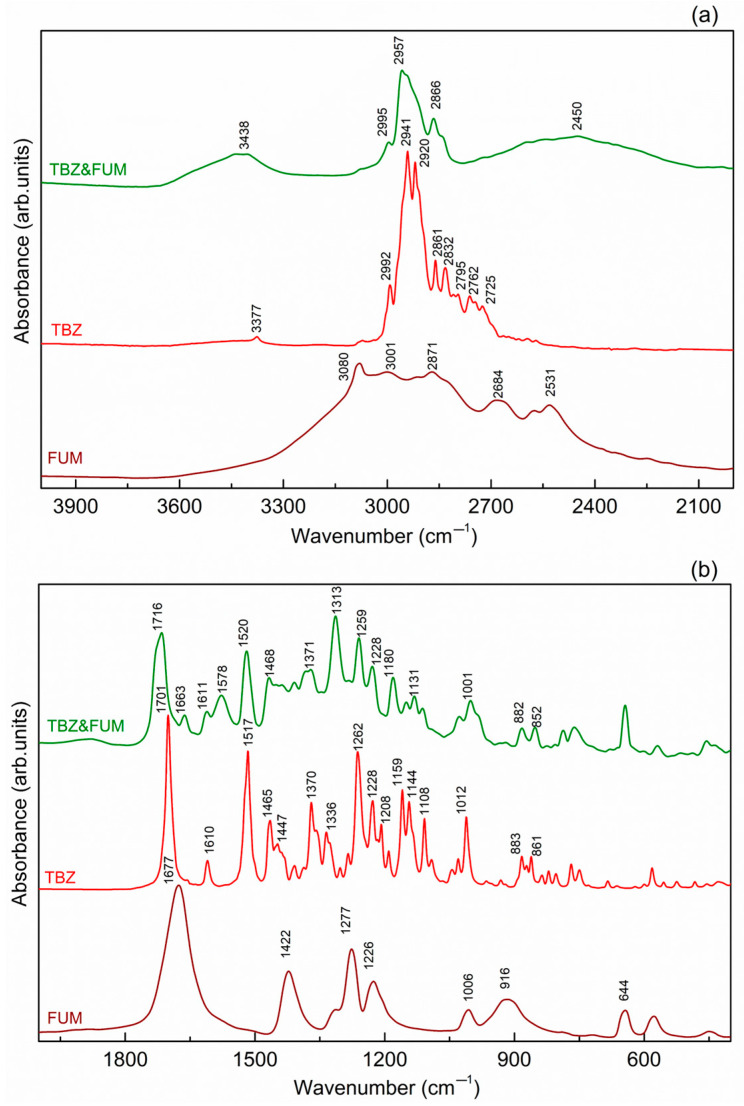
FTIR spectra comparison of fumaric acid, tetrabenazine, and the new fumarate salt of tetrabenazine, in the higher frequency region (**a**) and the fingerprint region (**b**), respectively.

The FTIR spectrum of fumaric acid (Figure 11) exhibits a very broad band, extending from 3350 cm^−1^ with a maximum at 3080 cm^−1^, which is attributed to the O-H stretching vibration of the carboxyl groups. Bands recorded between 3001 and 2531 cm^−1^ correspond to the C-H stretching vibrations. The carbonyl C=O group is identified by a broad band at 1677 cm^−1^.

In the spectrum of the new TBZ&FUM adduct (Figure 11), a broad band with a peak at 3438 cm^−1^ is observed, which is attributed to the O-H stretching vibration of the fumarate anion. The bands associated with C-H stretching vibrations are observed between 2995 and 2866 cm^−1^, originating from both the fumarate anion and the tetrabenazinium cation. The new broadband at 2450 cm^−1^ in the TBZ&FUM spectrum can be related to the N-H vibration of the protonated nitrogen atom in the tetrabenazinium cation, which is absent in the tetrabenazine spectrum.

The carbonyl group C=O vibrations from both the tetrabenazinium cation and the fumarate anion are observed at 1716 and 1663 cm^−1^, respectively. The C=C stretching vibrations are detected between 1611 and 1520 cm^−1^.

#### 3.5.3. TBZ&SUC

**Figure 12:**
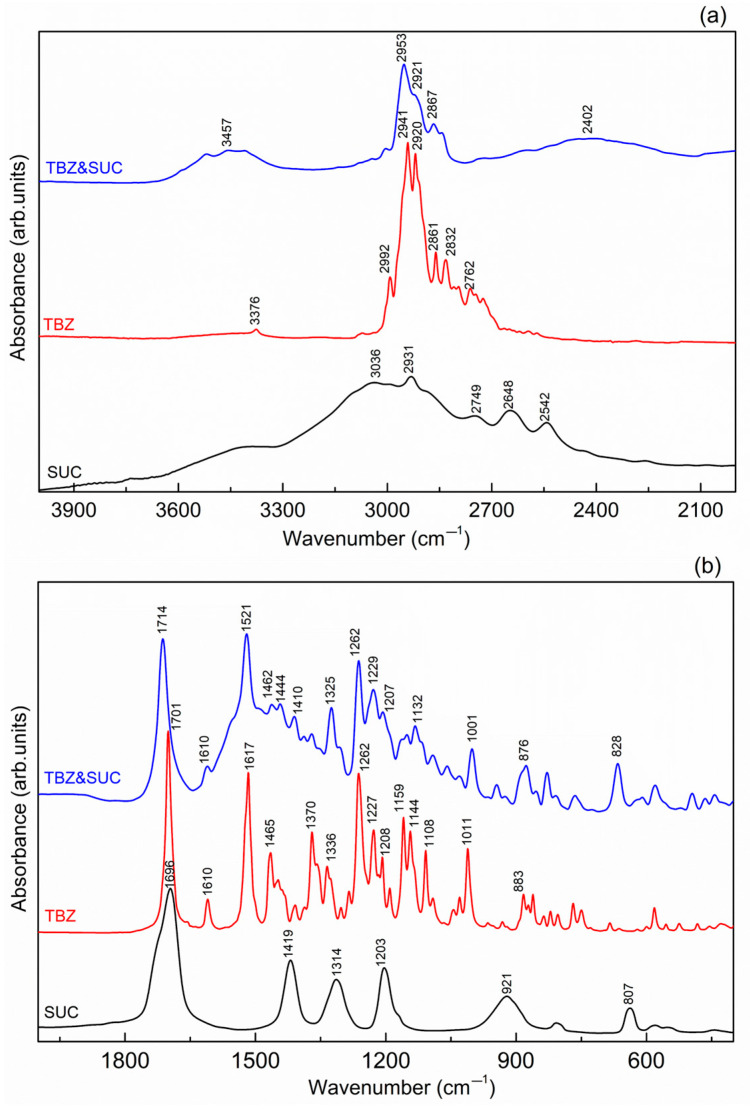
FTIR spectra comparison of succinic acid, tetrabenazine, and the new succinate salt of tetrabenazine, in the higher frequency region (**a**) and the fingerprint region (**b**), respectively.

The FTIR spectrum of succinic acid (Figure 12) shows a broad peak at approximately 3400 cm^−1^, which corresponds to the hydroxyl O-H stretching vibrations of the carboxyl groups. The bands found between 3036 and 2542 cm^−1^ are attributed to the C-H vibrations, while the C=O stretching vibration is observed at 1696 cm^−1^.

In the spectrum of the TBZ&SUC salt (Figure 12), the band at 3467 cm^−1^ is associated with the O-H vibration of the hydroxyl group in the succinate anion. The bands found between 2963 and 2867 cm^−1^ correspond to C-H vibrations. The broad peak at 2402 cm^−1^ is linked to the N-H stretching vibration of the protonated tetrabenazinium cation. The C=O stretching vibration is observed at 1714 cm^−1^, and the C=C stretching vibrations are found at 1610 and 1521 cm^−1^.

## 3.6. In Vitro Solubility Assessment

Stock solutions of the pure tetrabenazine and the new salts (C_TBZ_ = 0.0888 mg/mL, C_TBZ&OX_ = 4.3478 mg/mL, C_TBZ&FUM_ = 4.2853 mg/mL, and C_TBZ&SUC_ = 1.000 mg/mL) were analyzed using UV-VIS spectroscopy in the 190–500 nm range. From the analysis of the spectra, it was observed that, unlike tetrabenazine, all the new salts exhibited supersaturation, with their spectra exceeding the spectral limits. To determine the wavelength at which the absorbance was maximum, the stock solutions were diluted to various concentrations, and the UV-VIS spectra were recorded. The dilution concentrations and UV-VIS spectra are provided in Figures S1–S4 (Supporting Information).

The maximum absorbance wavelength was determined to be λ = 283 nm for all the compounds. Calibration curves were constructed for five different concentrations of the diluted solutions, using the 283 nm wavelength.

From the quantitative analysis based on these calibration curves, the concentrations of the diluted solutions were determined. A small difference between the experimental concentration (calculated from the initial amount of dissolved sample) and the concentration obtained from the filtered solutions was noted. This difference represents the mass of the substance lost during the filtration process (see Table S2, Supporting Information).

## 4. Conclusions

Three new salts of tetrabenazine, namely oxalate, fumarate, and succinate, were successfully synthesized and analyzed. Their crystal structures were determined, revealing that all three forms are centrosymmetric: the oxalate salt adopts a monoclinic structure, while the fumarate and succinate salts are triclinic. The formation of these salts involves the deprotonation of carboxylic acids and the migration of a hydrogen atom to the nitrogen of the tetrabenazine molecule. The supramolecular architectures are stabilized by N-H···O hydrogen bonding interactions between the host (tetrabenazine) and guest (carboxylate anion) molecules, being further complemented by C-H···O and O-H···O interactions.

X-ray powder diffraction analysis confirmed the purity of the newly prepared salts and demonstrated their stability under specific conditions, without undergoing phase transitions. DSC thermal analysis revealed that each salt has distinct melting points, differentiating them from the starting materials. Additionally, Fourier-transform infrared (FTIR) spectroscopy provided insight into the protonation of the nitrogen atom in the tetrabenazine molecule.

From a pharmaceutical perspective, UV-VIS spectroscopy indicated that the newly obtained salts exhibit significantly higher aqueous solubility compared to the original tetrabenazine form, suggesting potential improvements in bioavailability for therapeutic applications.

## Figures and Tables

**Table 1:** Single-crystal X-ray diffraction data of the tetrabenazine salts.

Crystal	TBZ&OX	TBZ&FUM	TBZ&SUC
Formula	C_19_H_28_NO_3_^+●^C_2_HO_4_^−^	C_19_H_28_NO_3_^+●^C_4_H_3_O_4_^−^	C_19_H_28_NO_3_^+●^C_4_H_5_O_4_^−^
D_calc_./g cm^−3^	1.290	1.268	1.259
µ/mm^−1^	0.802	0.773	0.766
Formula Weight	407.45	433.49	434.66
T/K	293(2)	293(2)	293(2)
Crystal System	monoclinic	triclinic	triclinic
Space Group	P2_1_/c	P-1	P-1
a/Å	5.6753(4)	6.3043(7)	10.0954(18)
b/Å	15.6643(8)	12.0998(10)	10.399(2)
c/Å	23.6427(17)	15.8475(12)	11.1625(15)
α/°	90	78.934(7)	87.063(15)
β/°	93.583(7)	79.640(8)	80.658(13)
γ/°	90	75.039(8)	82.728(17)
V/Å^3^	2097.7(2)	1135.30(18)	1146.5(4)
Z	4	2	2
Z’	1	1	1
Wavelength/Å	1.54184	1.54184	1.54184
Radiation type	Cu Kα	Cu Kα	Cu Kα
ϴ_min_/°	3.387	2.868	4.015
ϴ_max_/°	71.333	71.067	71.146
Measured Refl.	13,425	7172	7084
Independent Refl.	4027	4289	4315
Reflections with I > 2(I)	2603	2360	2479
R_int_	0.0433	0.0498	0.0530
Parameters	272	293	295
Restraints	0	3	0
Largest Peak	0.152	0.308	0.365
Deepest Hole	−0.171	−0.294	−0.332
GooF	1.182	1.030	1.047
wR_2_ (all data)	0.2186	0.2611	0.2791
wR_2_	0.1987	0.1954	0.2227
R_1_ (all data)	0.1013	0.1160	0.1196
R_1_	0.0624	0.0680	0.0821

## Data Availability

Data is contained within the article and Supplementary Materials. CIF files of the tetrabenazine-based crystals were deposited by the Cambridge Crystallographic Data Centre: 2433120 (TBZ&OX); 2433121 (TBZ&FUM), and 2433122 (TBZ&SUC). The files can be obtained free of charge on written application to CCDC, 12 Union Road, Cambridge, CB2 1EZ, UK (fax:+44 1223 336033); on request via e-mail to deposit@ccdc.cam.uk or by access to http://www.ccdc.cam.ac.uk (accessed on 14 May 2025).

## Supplementary Materials

The following supporting information can be downloaded at: https://www.mdpi.com/article/10.3390/pharmaceutics17050670/s1, Figure S1. UV-VIS spectra obtained for different concentrations of TBZ (a), and calibration curve graph of TBZ (b); Figure S2. UV-VIS spectra obtained for different concentrations of TBZ&OX (a), and calibration curve graph of TBZ&OX salt (b); Figure S3. UV-VIS spectra obtained for different concentrations of TBZ&FUM (a), and calibration curve graph of TBZ&FUM salt (b); Figure S4. UV-VIS spectra obtained for different concentrations of TBZ&SUC (a), and calibration curve graph of TBZ&SUC salt (b); Table S1. Intermolecular interactions involved in crystal packing (Å, °); Table S2. Quantitative analysis of calibration curves
