# Supplementary material for: Developing the Oxalate, Fumarate and Succinate Salts of Tetrabenazine: Solid-State Characterization and Solubility
Source: Pharmaceutics. 2025 May 20;17(5):670. doi: 10.3390/pharmaceutics17050670 (PMC12115168; doi:10.3390/pharmaceutics17050670)
Supplement: Supplementary file 1 [file pharmaceutics-17-00670-s001.zip › pharmaceutics-3579956-supplementary.pdf]

Supporting Information

Table S1. Intermolecular interactions involved in crystal packing (Å, °)

Structure	D-H···A	D-H	H···A	D···A	<(D-H···A)	Symmetry Operation
TBZ&OX	N1-H1···O4	0.996	1.75(3)	2.741(3)	173.1(3)	x,y,z
	C6-H6B···O7	0.970	2.580(2)	3.212(4)	122.92(17)	x,y,z
	C1-H1C···O7	0.960	2.674(3)	3.575(5)	156.4(4)	x,y,z
	C8-H8A···O5	0.970	2.403(2)	3.333(4)	160.5(1)	-x,-1/2+y,1/2+z
	C9-H9···O6	0.980	2.614(1)	3.388(3)	135.94(17)	1-x,-1/2+y,1/2-z
	C18-H18A···O6	0.960	2.652(3)	3.492(5)	146.3(1)	-x,-1/2+y,1/2-z
	C18-H18B···O3	0.960	2.562(3)	3.493(5)	163.3(5)	-1-x,1-y,1-z
	C6-H6B···O4	0.970	2.5179(19)	3.359(3)	145.03(19)	1+x,y,z
	O7-H7···O5	0.814	1.75(2)	2.56(3)	172.1(7)	1+x,y,z
TBZ&SUC	N1-H1···O4	0.994	1.688(19)	2.674(5)	171.5(3)	x,y,z
	C5-H5···O4	0.980	2.684(4)	3.405(6)	130.8(3)	x,y,z
	C6-H6B···O5	0.970	2.628(4)	3.233(7)	120.7(3)	x,y,z
	N1-H1···O5	0.994	2.587(2)	3.142(3	114.5(1)	x,y,z
	C1-H1B···O3	0.960	2.630(3)	3.574(8)	167.8(6)	1+x,y-1,-1+z
	C8-H8A···O1	0.970	2.586(4)	3.492(6)	155.4(3)	2-x,1-y,2-z
	C8-H8A···O2	0.970	2.652(3)	3.511(6)	147.8(2)	1-x,1-y,2-z
	C18-H18B···O4	0.959	2.517(3)	3.410(6)	154.9(3)	1-x,1-y,2-z
	C14-H14···O6	0.930	2.470(4)	3.381(6)	166.4(3)	1-x,2-y,1-z
	C8-H8B···O2	0.970	2.652(3)	3.511(6)	147.8(2)	1-x,1-y,2-z
	C9-H9···O6	0.980	2.525(4)	3.383(6)	146.1(3)	x,y,1+z
TBZ&FUM	N1-H1···O4	0.981	1.74(2)	2.711(3)	173.1(4)	x,y,z
	N1-H1···O5	0.981	2.39(4)	3.028(4)	122.1(3)	x,y,z
	C8-H8A···O4	0.971	2.647(2)	3.313(4)	126.10(19)	x,y,z
	C12-H12B···O7	0.971	2.526(3)	3.435(5)	156.1(3)	2-x,1-y,1-z
	C19-H19A···O6	0.960	2.656(3)	3.556(6)	156.3(4)	1+x,-1+y,z
	C13-H13B···O6	0.970	2.559(3)	3.232(5)	126.5(2)	1-x,1-y,1-z
	C18-H18C···O1	0.960	2.696(3)	3.596(7)	156.2(5)	2-x,1-y,-z
	C21-H21···O5	0.930	2.513(2)	3.190(4)	129.88(19)	1+x,y,z
	O7-H7···O5	0.915	1.634(15)	2.539(4)	173.1(4)	1+x,y,z
	C22-H22···O7	0.930	2.529(2)	3.199(4)	129.3(2)	-1+x,y,z

Table S2. Quantitative analysis of calibration curves.

TBZ		TBZ&OX		TBZ&FUM		TBZ&SUC	
**exp**	**calc**	**exp**	**calc**	**exp**	**calc**	**exp**	**calc**
0.0177	0.0204	0.0437	0.0534	0.0215	0.0339	0.025	0.0329
0.0355	0.0389	0.1304	0.1735	0.0430	0.0601	0.050	0.0515
0.0532	0.0534	0.2173	0.2361	0.0860	0.0960	0.100	0.0973
0.0710	0.0698	0.3260	0.3421	0.1290	0.1289	0.150	0.1442
0.0888	0.0873	0.4347	0.4006	0.1720	0.1596	0.200	0.2016

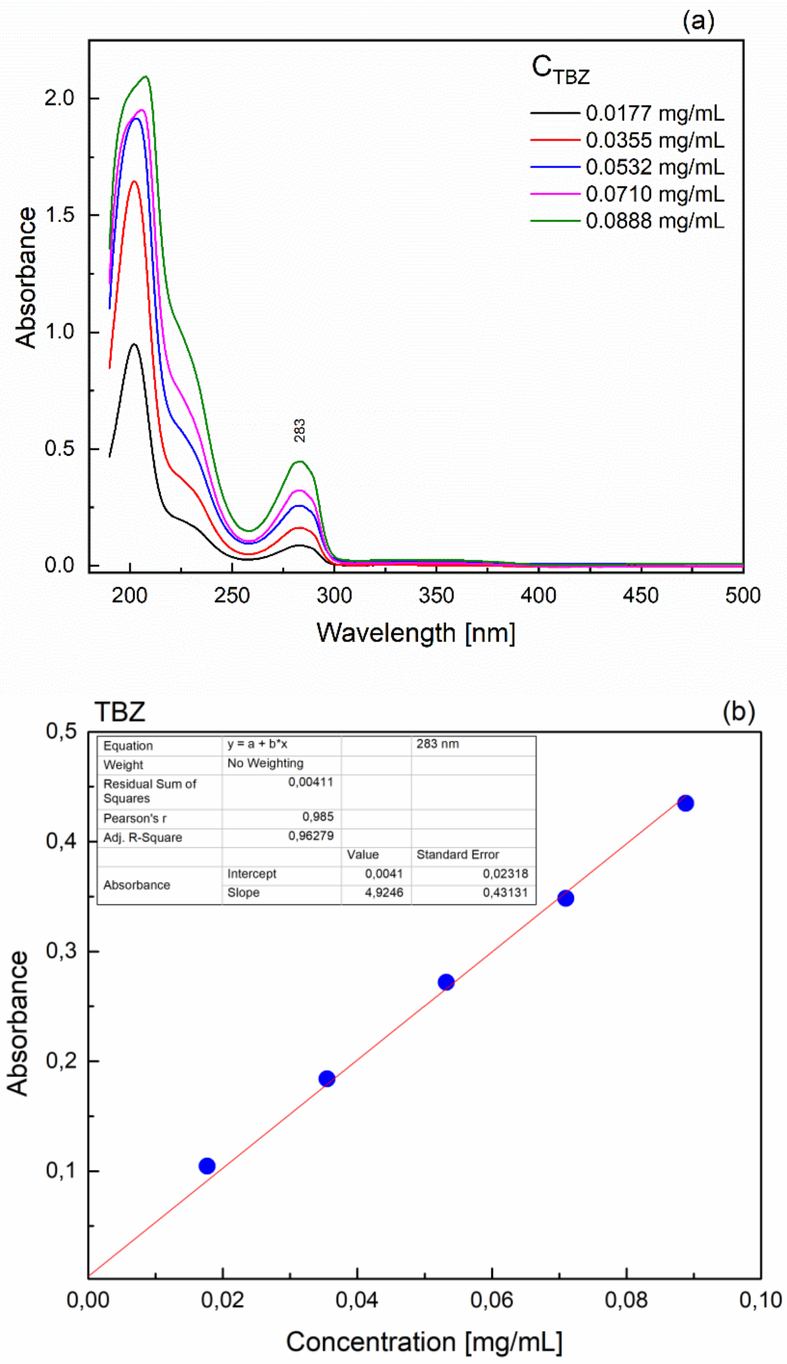

Figure S1. UV-VIS spectra obtained for different concentrations of TBZ (**a**), and calibration curve graph of TBZ (**b**).

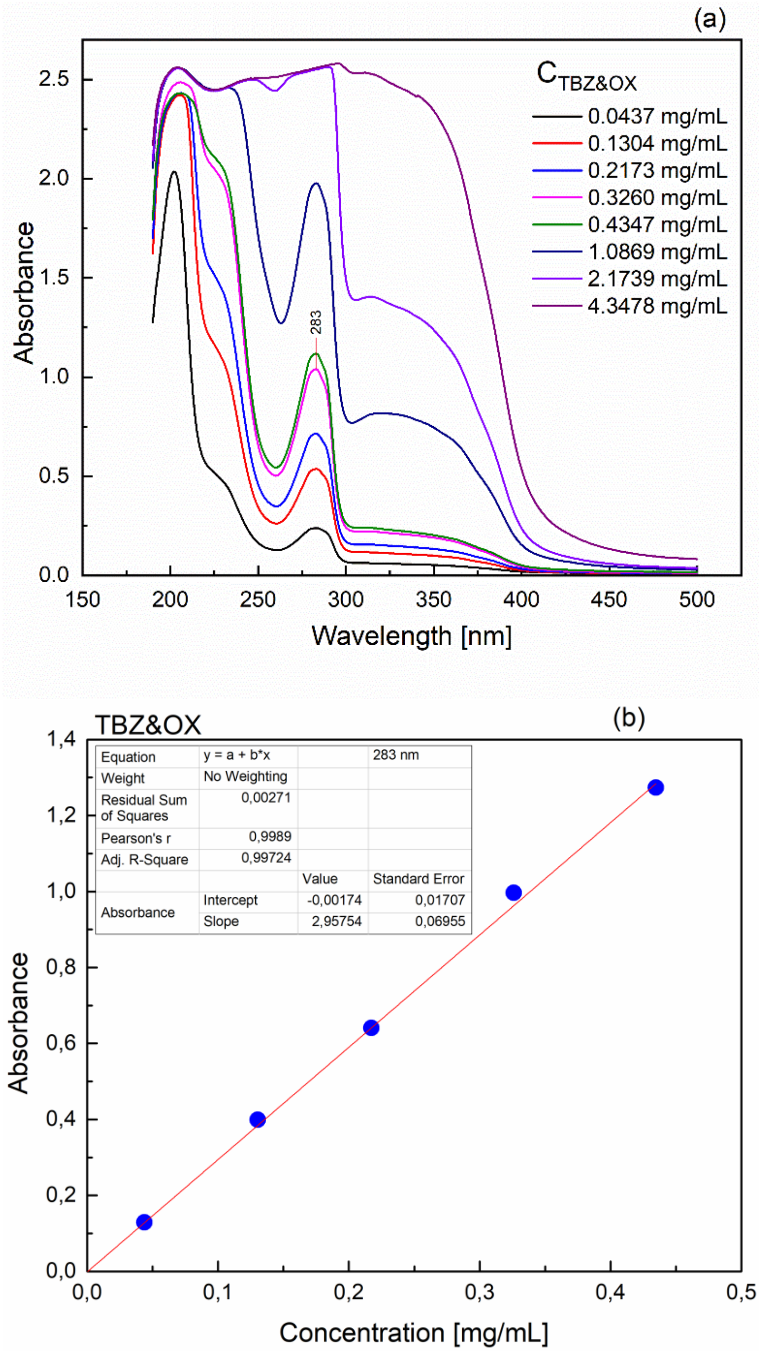

Figure S2. UV-VIS spectra obtained for different concentrations of TBZ&OX (**a**), and calibration curve graph of TBZ&OX salt (**b**).

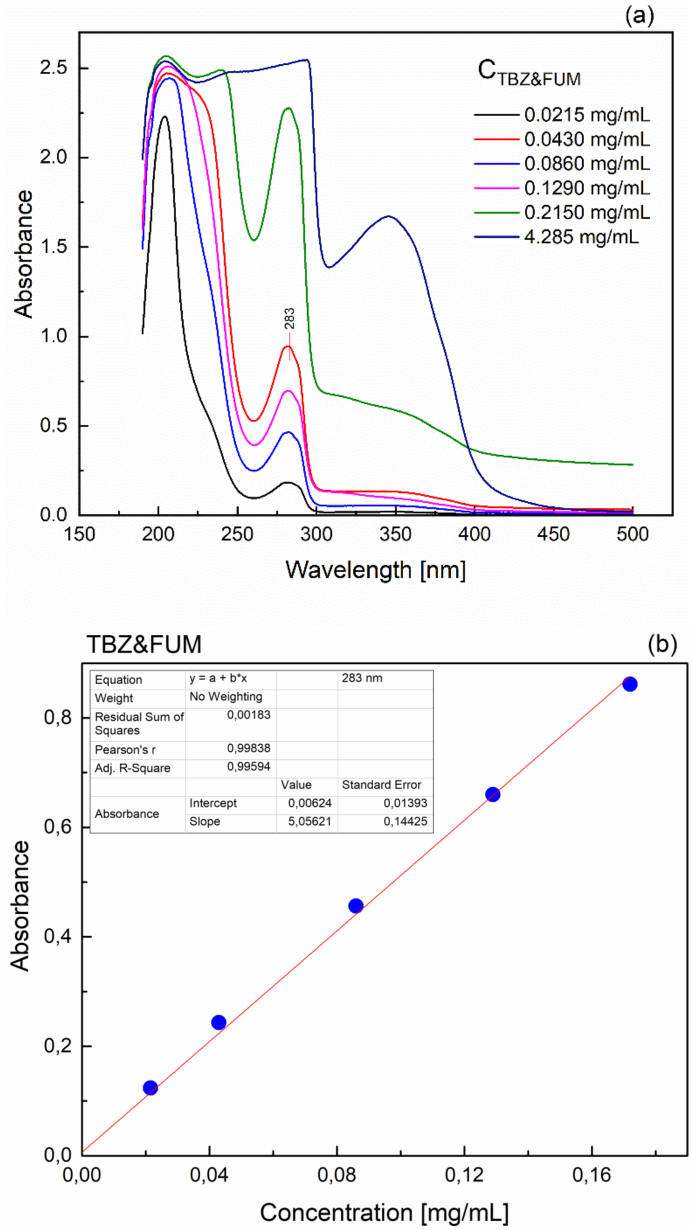

Figure S3. UV-VIS spectra obtained for different concentrations of TBZ&FUM (**a**), and calibration curve graph of TBZ&FUM salt (**b**).

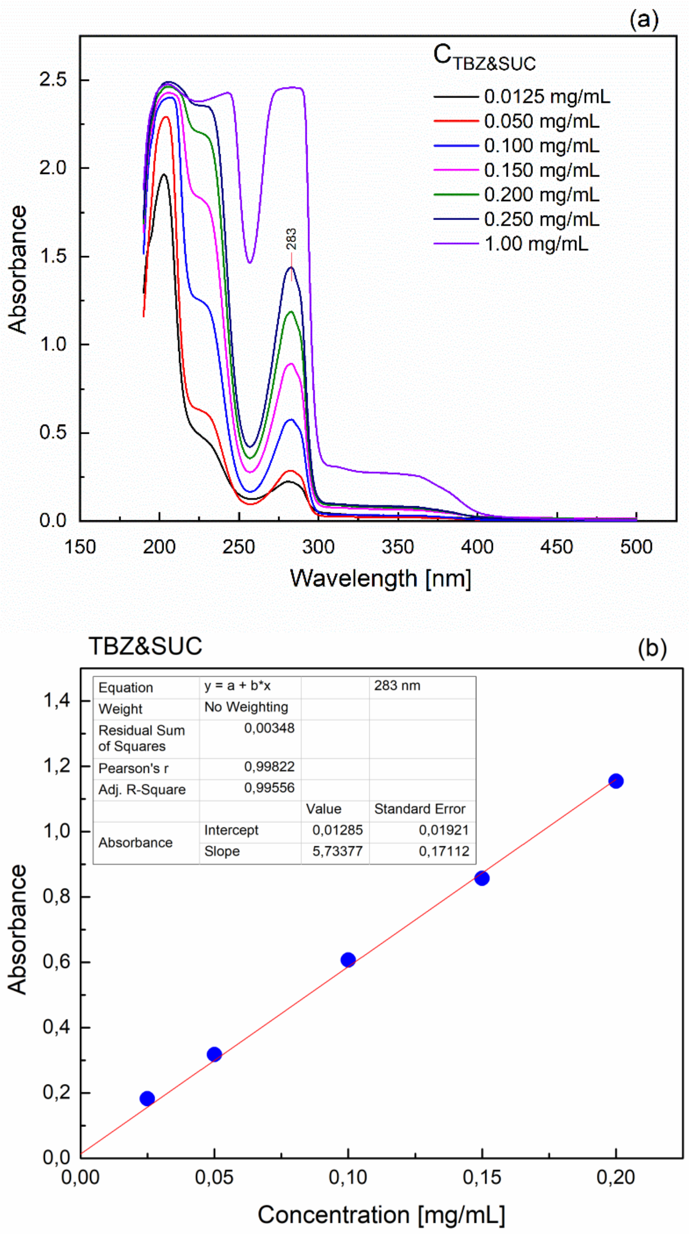

Figure S4. UV-VIS spectra obtained for different concentrations of TBZ&SUC (**a**), and calibration curve graph of TBZ&SUC salt (**b**).
